# Community Health Resources, Globalization, Trust in Science, and Voting as Predictors of COVID-19 Vaccination Rates: A Global Study with Implications for Vaccine Adherence

**DOI:** 10.3390/vaccines10081343

**Published:** 2022-08-18

**Authors:** Shadi Omidvar Tehrani, Douglas D. Perkins

**Affiliations:** Department of Human & Organizational Development, Peabody College, Vanderbilt University, Nashville, TN 37203, USA

**Keywords:** COVID-19, vaccination, vaccine hesitancy, community health resources, globalization, public attitudes, voter participation, government effectiveness, fiscal decentralization

## Abstract

The COVID-19 global pandemic requires, not only an adequate supply of, but public adherence to safe and effective vaccinations. This study analyzes the human and economic resources and political and public attitudinal factors that influence widely varying country-level coronavirus vaccination rates. Using data on up to 95 countries, we found that countries’ strength of community health training and research (CHTR), education index, globalization, and vaccine supply are associated with a greater COVID-19 vaccination rate. In a separate analysis, certain political factors, and public attitudes (perceived government effectiveness, government fiscal decentralization, trust in science, and parliamentary voter turnout) predicted vaccination rates. Perceived corruption and actual freedoms (political rights and civil liberties) related to vaccination rates in prior studies were not significantly predictive when controlling for the above factors. The results confirm our prior findings on the importance of CHTR resources for increasing COVID-19 vaccination rates. They also suggest that to motivate vaccine adherence countries need, not only an adequate vaccine supply (which depends on a country having either its own resources or effective global political, social, and economic connections) and community health workforce training and research, but also a population that trusts in science, and is actively engaged in the political process.

## 1. Introduction

The highly contagious SARS-CoV-2 virus created a global pandemic called coronavirus disease 2019 (COVID-19), which continues to pose great risks to countries [1]. In terms of severity, fatality, psychological health, and life expectancy, the COVID-19 outbreak has taken a tremendous toll on global wellbeing [2]. As new forms of SARS-CoV-2 continue to arise, the global population is threatened by yet another pandemic [3].

The development of the COVID-19 vaccine was deemed essential to control the recurring infection waves, reduce the death rate, and restore the pre-pandemic state of normalcy [4]. Various institutions and the World Health Organization (WHO) have collaborated to assist the vaccine manufacturers in developing and delivering COVID-19 vaccines effectively [5]. Mass immunization is required to increase herd immunity and guarantee that death rates are reduced [6]. However, vaccine hesitancy—the decision to postpone or refuse accessible vaccinations—is a significant barrier to the efficacy of continuing COVID-19 vaccination programs in many parts of the world [7]. Prior to this pandemic, the World Health Organization identified vaccination hesitancy as one of the top 10 health threats [8]. 

It is necessary for governments and non-governmental organizations to understand the reason behind the acceptance and reluctance of vaccines to create better measures to enhance COVID-19 vaccinations. To this aim, scholars have explored factors affecting the COVID-19 acceptability in a specific country or region [9]. However, to our knowledge, no study has identified the resource, trust, and political factors associated with vaccination acceptance and resistance across countries.

## 2. Resources

Community health workers (CHWs) have been vital to health and health systems for decades before the COVID-19 pandemic. CHWs offer culturally appropriate health knowledge and training, improve interactions between communities and healthcare institutions, advocate for individual and community demands, conduct outreach, and monitor and evaluate community health [10,11,12]. During the COVID-19 pandemic, CHWs play crucial roles in distributing new vaccines, including planning, targeting the different populations, community involvement and participation, and follow-up [13]. 

Many studies show that training in pandemic skills and knowledge enhance disease awareness, monitoring, and reporting [10,13,14]. However, the kind and extent of training for CHWs vary by country, which may influence vaccination rates [15]. Despite the importance of such training and education to the work and safety of CHWs, they are not always sufficient, frequent, and appropriate, reducing workforce efficiency in many countries [16]. Competency-based education and learning programs emphasizing the need to control the spread of COVID-19, immunizations, communication, and community engagement are key factors in preparing CHWs for vaccination campaigns [13].

The general education level of the public also influences vaccination attitudes and experiences, such as trust in the vaccine approval process and assessment of the severe side effect risks [17,18]. Higher levels of education are associated with increased health and vaccination literacy and awareness of the need for universal vaccination coverage [19,20]. Lazarus et al. [21] found that education was substantially associated with vaccine acceptance among respondents from the United States, Ecuador, France, Germany, and India. 

The economic harm caused by restriction policies intended to curb the spread of coronavirus has severely reduced household incomes, particularly for lower-income populations. Measures such as social distancing and lockdowns involve trade-offs between COVID-19 management approaches and their social and economic costs—such as social instability, poverty, and limitations on public services—which have downstream consequences on health outcomes not directly related to COVID-19. Low-income workers who disproportionately experienced job losses or income cutbacks are more likely to encounter financial hardship, such as difficulties obtaining food, paying medical expenditures, mortgage, rent, and bills [22,23,24]. 

Economic stress influences vaccine hesitancy, and this dilemma particularly affects developing nations with insufficient public welfare [25]. According to Kaiser Foundation surveys, low-income parents were concerned about vaccine-related costs, such as taking time off work and transportation. Government can increase the vaccination rate through pandemic-related income supports [26]. 

Just as governments have rightfully closed borders and offered economic assistance to safeguard their populations during the pandemic, they also have the right and duty to guarantee their citizens access to an effective vaccine [27]. Public health professionals used the phrase vaccine nationalism to describe governments that provide their people with vaccinations before delivering them to other nations [28]. Governments can achieve this objective with various methods, but the most common is to ensure a supply through agreements with pharmaceutical companies that prioritize their orders by giving a higher price or by negotiating during the pre-approval phase [29]. 

Countries that disapprove of the morality and efficacy of Advance Purchase Agreements or lack the financial means to buy vaccines at similar rates run the risk of delayed access when orders from wealthy countries fill manufacturing capacity first [30]. As the pandemic progressed, different industrialized nations, such as Japan, Canada, the US, and the UK, signed agreements with pharma firms to ensure future vaccine supply. By mid-January 2021, despite having just 14% of the world’s population, these nations had purchased 60% of the 7 billion vaccinations supplied [31]. 

On the other hand, many researchers have advocated for a more globally coordinated strategy, in which governments agree to forego their national interest in securing vaccines for their citizens and transfer the responsibility of distributing the vaccine internationally based on need. Netherlands, Germany, France, and Italy created the Inclusive Vaccine Alliance early in the outbreak to assure immunization availability for European nations and some low-income countries [32].

Globalization fosters growth via economic (movement of products, wealth, services, financial reports, and market views), social (dissemination of ideas, information, culture, and individuals), and political integration (diffusion of governance and participation in international coordination efforts) [33]. Many globalization-related factors have altered the delivery of vaccination services and the supply of vaccines, including greater worldwide investment, harmonization of regulatory requirements across borders, and manufacturing in developing countries [34]. 

### 2.1. Public Attitudes and Politics

Teremetskyi et al. [35] identified contemporary health issues that require investigation and commitment. These issues result from dishonest actions such as resource exploitation, deception in public contracts, misuse of finances, and medical supply theft. Regarding the pandemic, COVID-19 immunization deficits cause global corruption and delayed vaccination [36].

The trends of aid allocation from Western donors indicate that considerable funds continue to flow to corrupt nations. Considering donor assistance policies that emphasize positive recipient performance, corrupt leaders are incentivized to adhere deliberately to donor objectives [37]. However, studies reveal that corrupt governments exploit the assistance to offer little health service sector coverage and appease funders with limited advancements in public health while justifying increased flows to profitable industries in order to gain wealth without any reciprocal contribution of productivity. Such corruption is evident in the pattern of immunization coverage in response to health aid flows [37,38]. Sommer [39] argues that corruption in the health sector reduces available money, increases patient expenditures, restricts service expansion, and lowers the quality of treatment. In addition, corruption undermines the government’s effectiveness in developing and implementing public programs and projects. Government effectiveness is measured by the quality of public services, policy design, application, the government’s adherence to such initiatives, and the independence of the civil service from political influences [40]. 

Implementation of large-scale health policies, such as mass immunizations, is dependent on successful governance, which integrates local, and urban organizations to deliver immediate and effective community strategies [41,42]. Effective governments may respond to the COVID-19 pandemic with integrated preparation, such as expanded testing and hospital facilities, monitoring, and distributing personal protective equipment and vaccines [43]. Dye et al. [44] revealed that enhancing government effectiveness is essential, as vaccination coverage is only statistically significant in nations with solid government effectiveness. Governments with greater effectiveness may be able to target high-risk individuals and provide immunizations to maximize community health [45]. 

However, vaccination is a complicated interaction of personal factors relating to the willingness and ability to get a vaccination, along with structural-systemic factors limiting its accessibility and availability [46]. Community awareness, trust in science to develop a safe and effective vaccine, and trust in the healthcare system to provide it are essential to vaccination acceptance [47]. 

Trust in science is explored concerning evidence-based preventative guidelines, such as mask-wearing, social distancing, handwashing, and immunization. Trust in science and experts is important for compliance with preventive measures because government requirements and restrictions are viewed as expert recommendations, and the effectiveness of these measures depends on individual cooperation [48,49]. Nevertheless, religion and ideology frequently undermine trust in science, which impairs public health behavioral compliance [15]. Plohl and Musil [50] found that political conservatives, religious orthodoxy, and conspiracy theorists have less confidence in science and are less likely to take precautions and get vaccinated. Unwavering faith in God, supported by the conviction that God would protect His believers from harm, is sometimes used as a rationale for their lack of confidence in science [51]. In addition, conspiracy theories about COVID-19 are associated with less scientific knowledge and dissemination of misinformation on the severity, treatment, and existence of coronavirus [52,53,54]. For example, since the outbreak’s onset, there has been significant suspicion that COVID-19, similar to other respiratory viral diseases, may display seasonality [55,56]. Even though research found negative outcomes and urged policymakers not to adapt interventions based on weather [57,58], there have been policy outcomes that study findings have explicitly advised against, resulting in public confusion regarding scientific precision or ambiguity and conspiracy theories [59].

According to proponents, government decentralization is intended to provide decision-makers with accurate, up-to-date information on the preferences and concerns of residents, allowing them to communicate their needs and objectives [60]. Fiscal decentralization distributes fiscal obligations from the central government to sub-national or local governments to improve public sector delivery [61]. A well-designed and implemented fiscal decentralization strategy is expected to increase equality, effectiveness, reliability, and coverage of health care services and health outcomes [62]. 

Numerous studies concentrate on decentralization and immunization rates. Ebel and Yilmaz [63] established a correlation between decentralization and measles vaccination coverage in six developing nations from 1970 to 1999. From 1980 to 1997, Khaleghian [60] examined fiscal decentralization and DPT3 and measles vaccination rates in 140 low- and middle-income countries. According to the research results, this may indicate a healthy balance between local officials’ connection to the public, central authority, and bureaucratic independence, all of which are critical for the success of an immunization campaign [60].

Major crises inspire debate over whether the government should limit personal liberties for the common good [64]. Anti-vaccine attitudes and lobbying are not new, but many believe that during the COVID-19 epidemic, coordinated efforts to induce an aversion to immunizations have increased [65]. Whether intentionally or unconsciously, anti-vaccinators combine freedom of choice with widespread skepticism of trust in authority and science. The notions of civil liberties and human rights are different yet interwoven. Both pillars of society involve the right not to be harmed by others. According to pro-vaccine advocates, herd immunity is the most effective way for individuals to protect themselves and one another from a potentially fatal infection [66]. Somewhat paradoxically, vaccination requirements increase civil liberties by avoiding mass closures, lockdowns, travel restrictions, and other stringent public health measures [15]. They protect people with disabilities and weakened immune systems, young children, and communities of color severely affected by the disease. By protecting individuals against the disease’s most severe symptoms, immunizations have the potential to restore their most fundamental rights, enabling them to resume their normal lives [67]. 

The most recent research reveals that people understand and respond differently to the information regarding the risk of a viral infectious disease or the related policy initiatives based on their ideology and political perspective [68,69]. 

Countries can predict compliance with COVID-19 restrictions and immunization based on pre-crisis social responsibility, such as voter turnout [70]. There is a negative relationship between voting behavior and vaccination hesitancy. Vaccine reluctance might be viewed as a sign of a more fundamental problem, namely a rejection of government or state action motivated by skepticism and distrust among people [71]. According to a study conducted in Australia, those who vote are more hopeful, have a greater quality of life, use credible sources of information, attempt to prevent viral infections, and are more accepting of vaccines [69]. 

### 2.2. Hypotheses 

In this study, we aim to identify economic, political, attitudinal, and public health-related factors which predict COVID-19 vaccination rates in 95 countries (see Supplementary Table S1). We hypothesized that the vaccination rate would be positively associated with certain resources—such as the economic support index, the extent of community health expertise, nurses per 10,000 population, population education index, globalization, and vaccine supply—and political and attitudinal factors—including trust in science, Freedom Score (civil liberties and political rights), (low) corruption perception, government effectiveness, fiscal decentralization, and parliamentary voter turnout in a country.

## 3. Methods

### 3.1. Data Sources

The study evaluated cross-sectional and panel studies’ secondary data. The country’s number of fully vaccinated individuals, resource and trust, and political indicators were gathered from different sources, such as Freedom House, the World Bank (WB), the World Health Organization (WHO), Gallup Poll, United Nations Development Program (UNDP), Transparency International, and the database of the Global Development of Applied Community Studies (GDACS) project of country-level education, economic, political, and social variables and the estimated strength of 12 community-focused research and professional disciplines in 105 countries.

### 3.2. Dependent Variable

On 17 May 2022, current World Health Organization (WHO) data on the number of fully vaccinated persons per 100 was obtained for 96 nations [72]. These data are gathered from government sources such as government reports, health institutions, and authorized press and are widely utilized by the media, legislators, academia, and the broader population to compare the effectiveness of national vaccination programs [73].

### 3.3. Resources Model Predictor Variables 

*Economic support index***.** The index tracks initiatives such as debt relief (i.e., if the government is suspending payment commitments for households) and income support (i.e., if the government offers direct payments to individuals who have lost their jobs or are unable to work) in 2022. This indicator is computed using all ordinal variables of economic policy. The range of the index is 0 to 100. A greater score implies better government support [74,75].

*Community health training and research.* We used the GDACS project, which is a dataset from 2016 to 2019 that used the internet and publications to describe undergraduate and graduate courses, training programs, journals, conferences, papers, and professional organizations to determine skilled experts and human research resources in the community public health [76]. GDACS database covers 105 countries representing over 95% of the world’s population. 

*Community health workers estimate.* We utilized the most updated data from 2022 WHO’s projection of community nurses and midwives (per 10,000 population) to quantify the CHWs [77].

*Education Index.* The latest education index in 2019 was gathered as a factor of the Human Development Index published by the UNDP annually. Beginning in 2010, the education index integrates average adult years of schooling with predicted years of schooling for those under 25 years old. [78].

*Globalization Index.* The KOF Globalization Index collected in 2021 is a scale of 43 factors evaluating globalization along the economic, social, and political dimensions for every nation on a scale from 1 (least) to 100 (most globalized). The global KOF Globalization Index is the mean of the de facto and de jure indices. Once the weights have been calculated, the aggregation includes the addition of individual weighted variables as opposed to the use of aggregated lower-level indicators. [79,80]. 

*Secured and/or Expected Vaccine Supply in total doses (% of the population).* Secured doses (excluding any optional doses on contracts) through bilateral agreements between countries and areas and vaccine manufacturers, collected through the COVAX Global Market Assessment by BMGF, CEPI, Gavi, PAHO Revolving Fund, UNICEF, and WHO, largely leveraging public sources of information [81]. 

### 3.4. Attitudes and Politics Model Predictor Variables

*Government Effectiveness.* The World Bank Group’s government effectiveness index in 2020 examines the quality of policy development, civil service, policy execution, public services, and the reliability of a government’s commitment to increase or maintain these qualities on a scale of −2.5 (poor) to 2.5 (strong) [82].

*Fiscal Decentralization.* We employed the World Bank’s fiscal decentralization index to measure variables such as the degree of fiscal autonomy of local governments in a country, local governments’ taxing policies, use of formula-based or unrestricted funds and transfers, and expenditure and borrowing freedom from external resources [83].

*Trust in Science.* The Wellcome Global Monitor study was conducted in 2020 and early 2021 as part of the Gallup World Poll that explores how the pandemic has affected people’s lives in 113 countries and territories and influenced their perceptions of science, healthcare systems, and governments. The proportion of participants who responded “a lot” or “some” to the question: “How much do you trust science?” was considered in this study [84].

*Corruption Perception.* The Corruption Perceptions Index measures public sector corruption perceptions, including political and administrative corruption. The index is generated on a scale from 0 (most corrupt) to 100 (least corrupt) using data from surveys and evaluations of corruption gathered by a range of credible agencies in 2021 [85].

*Freedom Score.* Freedom House evaluates individuals’ civil liberties and political rights in 210 nations and territories annually [86,87]. The Freedom Score in 2021 is calculated by averaging the separate 0–10 ratings for political rights and civil freedoms, with higher scores signifying higher freedom.

*Parliamentary Voter Turnout.* We applied the global voter database of the Institute for Democracy and Electoral Assistance to assess parliamentary voter turnout from 2009 to 2015. Using just parliamentary voter participation statistics is justified since many nations elect their prime ministers through parliament. This is a percentage of the voting-age population, not registered voters. Because deaths or movements of electors from one district to another are not recorded in the roll, a common problem for election officials worldwide makes it difficult to maintain an accurate voter roster [88].

## 4. Analysis

Bivariate correlations and hierarchical multiple linear regression analyses were used to evaluate the relationship between country-level characteristics and vaccination rates per 100 individuals. Initially, descriptive statistics (mean, standard deviation) are employed to better comprehend the data characteristics pertinent to this investigation (see Table 1). 

We then investigated the bivariate linear associations of each set of independent variables (resources in Table 2; attitudes and politics in Table 3) among each other and with the outcome variable (COVID-19 vaccination rate) using separate Pearson correlation matrices.

After excluding predictors in order to reduce multicollinearity and suppression effects, hierarchical multiple linear regressions were used to estimate both the accumulated and unique influence of two sets of predictor variables on the vaccination rate. First, in a set of resource-related models (Table 4), the first step of the hierarchical regression includes economic and health resource variables such as the economic support index, the strength of CHTR, and the quantity of nursing and midwifery workers (per 10,000). The second step included either (due to their high intercorrelation) education index (Model 2) or globalization index (Model 4). The third step (final models 3 and 5) added secured and/or expected vaccine supply, in order to show both how much it contributes significantly beyond the other predictors, but also, if the supply were not a factor, which predictors in the prior models would be significant.

Another set of multiple regressions was conducted to model the influence of public attitudes and politics on COVID-19 vaccination rates, with fiscal decentralization, government effectiveness, and trust in science added in the first step and corruption perception index, freedom parliamentary voter turnout included in the latter (Table 5). The analysis was performed at the two-tailed p.05 significant level.

## 5. Results

Supporting our hypotheses, simple bivariate Pearson correlation analysis for the resource model showed significant positive country-level correlations (*n* = 95) between persons fully vaccinated per 100 people and economic support index (*r* = 0.374, *p* < 0.001), CHTR (*r* = 0.485, *p* < 0.001), nursing and midwifery personnel (per 10,000) (*r* = 0.518, *p* < 0.001), education index (*r* = 0.717, *p* < 0.001), globalization index (*r* = 0.708, *p* < 0.001), and secured and/or expected vaccine supply in total does (*r* = 0.660, *p* < 0.001) (see Table 2). In the attitudes and politics correlation matrix (Table 3; *n* = 74), significant bivariate predictors of vaccination rate include government effectiveness (*r* = 0.672, *p* < 0.001), fiscal decentralization index (*r* = 0.625, *p* < 0.001), trust in science (*r* = 0.599, *p* < 0.001), corruption perception index (*r =* −0.569, *p* < 0.001), freedom score (*r* = 0.474, *p* < 0.001), and parliamentary voter turnout (*r* = 0.546, *p* < 0.001).

Next, two sets of hierarchical multiple linear regression models separately analyzed the influence of various health and economic resources and a set that modeled the influence of public attitudes and politics on the vaccination rate in each country. In the first set of resource models (Table 4; *n* = 95), the first step was statistically significant (R^2^ = 0.427, F (2, 91) = 22.80; *p* < 0.001). In step two, the R-squared value (R^2^ = 0.549, F (1, 90) = 24.35; *p* < 0.001) explained an additional 12.2% of variance in the vaccination rate by including education index. Finally, the addition of secured and/or expected vaccine supply to the regression model explained an additional 2.9% of the variation in COVID-19 immunization rate, and this change (R^2^ = 0.578, F (1, 89) = 6.15, *p* < 0.05) was also significant. In the final model, CHTR (β = 0.171; *p* < 0.05), education index (β = 0.437; *p* < 0.01), and secured and/or expected vaccine supply (β = 0.269; *p* < 0.05) each uniquely and significantly predicted a country’s vaccination rate. 

In the last two resource models, the globalization index replaced the education index in step two of the regression. Globalization index contributed significantly to the regression model (R^2^ = 0.527, F (1, 90) = 22.80; *p* < 0.001) and accounted for an additional 10% of the variation in vaccination rate. Adding secured and/or expected vaccine supply to the final step of the model (R^2^ = 0.548, F (1, 89) = 4.16; *p* < 0.05) explained an additional 2.1% of the variation. The final model showed that strength of CHTR (β = 0.181; *p* < 0.05), globalization index (β = 0.373; *p* < 0.01), and secured and/or expected vaccine supply (β = 0.254; *p* < 0.05) accounted for 54.8% of the variance in predicting COVID-19 vaccine rate. 

Finally, another set of hierarchical multiple linear regression models was calculated for public attitudes and politics predictors of country-level COVID-19 vaccination rates (Table 5, *n* = 74). The results of the first step of the regression indicated the three predictors explained 54.1% of the variance (R^2^ = < 0.541, F (3,70) = 27.50, *p* < 0.001). It was found that vaccination rate is significantly predicted by government effectiveness (β = 0.303, *p* < 0.05), fiscal decentralization (β = 264, *p* < 0.05), and trust in science (β = 0.290, *p* < 0.05). The last step showed that adding freedom score, corruption perception index, and parliamentary voter turnout added significantly to the model predicting vaccine rate (R^2^ = 0.633, F (3, 67) = 22.80; *p* < 0.01), explaining an additional 9.2% of the variance. The individual predictors were examined further and indicated that parliamentary voter turnout (β = 0.321; *p* < 0.001) was a significant predictor in the model while freedom score and corruption perception were not. 

## 6. Discussion

The coronavirus pandemic has posed an enormous burden and ongoing risk to global public health [2]. Countries have implemented a variety of tactics to prevent the spread of the illness, but as most countries have failed to keep infection rates low, constantly evolving COVID-19 variants have allowed the pandemic to continue indefinitely [89,90,91]. The present pandemic crisis is centered on the need to reduce the disease’s spread through global vaccine distribution. Vaccination acceptance is essential for herd immunity, yet vaccine hesitancy in many countries impedes attaining this end [15]. 

This is the first study to model COVID-19 vaccination rates that considers resources, public attitudes, and politics based on a sample of up to 95 nations worldwide. Our results suggest that resources such as the strength of CHTR, education, globalization, and a country’s secured vaccine supply are associated with a greater COVID-19 vaccination rate. 

We found that CHTR and COVID-19 vaccination rates are highly associated. Community health workers have always been an integral part of efforts to control the spread of HIV/AIDS, tuberculosis, and malaria [92] through community engagement, education, and preventative measures [93]. Community health practitioners possess the adaptable and relevant skills necessary across the COVID-19 disease continuum, from preventing SARS-CoV-2 viral transmission to reducing the severity of COVID-19 and its health, social, and economic implications [94]. With racial, ethnic, and language congruence, CHWs can target COVID-19-risk populations, increase the acceptability and uptake of vaccinations, and share experiences that promote community confidence and commitment [13]. 

One of our hypotheses explores each nation’s CHTR to explain this relationship. COVID-19 training for CHWs differs by area and country. Various studies have determined that CHW awareness, monitoring, and reporting may be enhanced by disease outbreak knowledge and skills [16,95,96]. Evidence-based treatments for outcome assessments are guided by factors such as years of experience and level of education [14]. In several trials, CHW training and education improved disease prevention, control, and vaccination rates. Through adequate training and education, CHWs can handle anxiety, build confidence in providing care, solve problems, and improve their communication and leadership abilities [97,98,99]. 

Our finding is in line with prior studies showing that a lower level of education results in reduced vaccination rate [100,101,102,103]. According to a nationally representative survey in the U.S., education has a great impact on individual’s willingness to receive vaccination. Those with a bachelor’s degree estimate the COVID-19 risk of a vaccinated individual to be 23% and vaccine adverse effects at 15%. In contrast, those with less education estimate the risk and side effects to be 34% and 31%, respectively [17]. 

According to the findings of this study, a secure vaccine supply is one of the critical factors in a greater vaccination rate. The effect of legal health determinants on the COVID-19 pandemic is evident when the law is used to support or hinder equitable access to COVID-19 immunizations worldwide. Vaccine nationalism leads to unequal access, as governments aim to exploit the legislation to gain early access to potential vaccines via Advance Purchase Agreements with vaccine makers [104]. As a result, countries with guaranteed supply and better access have a higher rate of vaccination. 

Conversely, globalization is anticipated to impact several aspects of public health, including infectious disease prevention by vaccination. COVID-19 triggered a global commitment of scientists and an unprecedented degree of international cooperation and creativity to find a vaccine [105]. Our result is consistent with previous research indicating a positive correlation between globalization and immunization rates. Globalization expedited the emergence of collaboration between developed and developing nations and increased the need for health policy, resulting in agreements that have impacted the supply and delivery of vaccines [34].

The present study also explains vaccination acceptance by political and attitudinal factors, including government effectiveness, fiscal decentralization, trust in science, and parliamentary voter turnout. Perceived corruption and actual freedoms (political rights and civil liberties) related to vaccination rates in prior studies [15] were not significantly predictive when controlling for the above factors. 

According to our findings, adopting large-scale health efforts, such as mass vaccinations, requires government effectiveness and fiscal decentralization. Vaccine-related challenges are usually cited as reasons for government effectiveness in funding and distribution [44]. The government purchases, delivers, implements, and promotes immunization in most countries where COVID-19 vaccination is offered as a public service. Therefore, government effectiveness shows the capability and preparedness to conduct COVID-19 vaccination programs [46]. In addition, fiscal decentralization would increase authorities’ commitment to local constituents and clarity, enabling a better allocation of goods and services to local needs [106]. Local decision-makers spend more on public health than on centralized government. In order to boost the vaccination rate, they may tailor staff and protocols to the local situation rather than relying on centralized approaches [60].

Science, government, and public health systems that supply and encourage vaccine uptake, promote accessibility and confidence [44]. We discovered that trust in science is significantly correlated with COVID-19 immunization. In preventative behavior, trust in science decreases approval of epistemic conspiracy conceptions and directly impacts complying with the measures and the willingness to get the COVID-19 vaccine [48,49,50]. Belief in the vaccine’s safety is a strong predictor of vaccination intent. To have confidence in a vaccine’s safety, one must trust the science behind its production, clinical studies, and quality standards [107]. 

Based on our findings, parliamentary voter turnout is a significant predictor of immunization rate in a country. There is evidence that a person’s worldview and political beliefs influence their adherence to COVID-19 restrictions, vaccine uptake, and government policy actions [68]. 

Historically, voter participation has been primarily explained by a feeling of civic responsibility. Voters derive internal satisfaction from conforming to a behavioral norm, and extrinsic rewards when others perceive them doing so. Social pressure is another aspect that motivates individuals to vote [108]. Thus, voting as a social obligation predicts compliance with other policy interventions, such as vaccination [70].

## 7. Conclusions

Despite being acknowledged as one of the most effective public health strategies, an increasing percentage of individuals consider vaccination harmful and unnecessary [109,110]. It is claimed that vaccine reluctance contributes to declining vaccination rates and an increased risk of vaccine-preventable disease epidemics. 

This study indicates that individual vaccination decisions are complicated and involve different resources, attitudes, and political factors. COVID-19 vaccine acceptance results from increased individuals’ trust in science, government health policies, and the policymakers’ effective methods, leading to a greater understanding of receiving the COVID-19 vaccine benefits. This research indicates the necessity for a variety of targeted approaches to reach vaccine-resistant individuals. Focused and tailored public health initiatives that increase public trust in vaccinations and underline the threat and severity of COVID-19 have the potential to reduce COVID-19 vaccine hesitancy and achieve herd immunity. Resources affecting the immunization rate include global collaboration between nations, vaccine supplies, high levels of education, and a community health workforce serving as a cultural liaison between communities and broader medical and public health systems. Public health interventions tailored to hard-to-reach populations are required to promote vaccine rate. Reliable sources with accurate information about vaccines, including language suitable for the wider public should be the primary goal of the healthcare professionals. 

This study highlights the need for strategies and initiatives that have been developed to increase vaccination acceptability as a result of the growing interest in vaccine hesitancy, such as openness in policy-making decisions regarding vaccination programs, enhancing confidence in the safety and efficacy of the COVID-19 vaccines, educating the community and health providers about the thorough processes that lead to the approval of new vaccines, and expanding vaccination access at the community level through different services.

## Figures and Tables

**Table 1:** Descriptive statistics.

	N	Mean	Std. Deviation
Cumulative number of persons fully vaccinated per 100 population	95	51.00	27.64
Economic support index	95	25.92	30.74
Nursing and midwifery personnel (per 10,000)	95	41.17	45.63
Current strength of Public Health	95	7.60	2.99
Education Index 2019	95	0.66	0.18
Globalization Index	95	64.98	14.21
Secured and/or Expected Vaccine Supply in total doses (% of population)	95	317.13	215.64
Government effectiveness	74	0.10	0.96
Fiscal Decentralization Index	74	0.42	0.22
Trust science	74	77.08	13.97
Corruption perception index	74	55.55	19.15
Freedom score	74	57.82	28.58
Parliamentary Voter Turnout 2016–2020 (%VAP)	74	0.60	0.16

**Table 2:** Correlation between country-level resource predictors and vaccination rate.

Pearson Correlation	1	2	3	4	5	6	7
1. Cumulative number of persons fully vaccinated per 100 population							
2. Economic support index	0.374 **						
3. Current strength of Public Health	0.485 **	0.211 *					
4. Nursing/midwifery personnel (per 10,000)	0.518 **	0.254 *	0.306 **				
5. Education Index 2019	0.717 **	0.414 **	0.472 **	0.705 **			
6. Globalization Index	0.708 **	0.486 **	0.505 **	0.703 **	0.860 **		
7. Secured and/or Expected Vaccine Supply in total doses (% of population)	0.660 **	0.407 **	0.390 **	0.651 **	0.738 **	0.811 **	

* *p* < 0.5, ** *p* < 0.01.

**Table 3:** Correlation between country-level political and attitudinal predictors and vaccination rate.

Pearson Correlation	1	2	3	4	5	6	7
1—Cumulative number of persons fully vaccinated per 100 population							
2—Government effectiveness	0.668 **						
3—Fiscal Decentralization Index	0.623 **	0.719 **					
4—Trust science	0.601 **	0.603 **	0.485 **				
5—Corruption perception index	−0.558 **	−0.781 **	−0.621 **	−0.530 **			
6—Freedom Score	0.467 **	0.544 **	0.470 **	0.301 **	−0.544 **		
7—Parliamentary Voter Turnout 2016–2020 (%VAP)	0.552 **	0.299 **	0.402 **	0.364 **	−0.17	0.176	

** *p* < 0.01.

**Table 4:** Summary of linear regression analysis for resource factors predicting COVID-19 vaccination rate (N = 95).

	Model 1			Model 2			Model 3			Model 4			Model 5		
Variable	B	SE B	β	B	SE B	β	B	SE B	β	B	SE B	β	B	SE B	β
Economic support index	0.191	0.075	0.212 *	0.082	0.07	0.092	0.05	0.069	0.056	0.049	0.075	0.054	0.041	0.074	0.045
Strength of community health training and research	3.042	0.778	0.329 ***	1.736	0.743	0.188 *	1.585	0.725	0.171 *	1.639	0.78	0.177 *	1.67	0.767	0.181 *
Nursing and midwifery personnel (per 10,000)	0.22	0.052	0.363 ***	0.025	0.061	0.042	−0.019	0.062	−0.032	0.04	0.063	0.066	0.014	0.063	0.024
Education index				83.962	17.013	0.561 ***	65.421	18.156	0.437 **						
Globalization index										1.062	0.243	0.546 ***	0.725	0.291	0.373 *
Secured and/or expected vaccine supply in total doses (% of population)							0.034	0.014	0.269 *				0.033	0.016	0.254 *
R2	0.427			0.549			0.554			0.527			0.548		
F for change in R2	22.804 ***			24.356 ***			6.153 *			19.037 ***			4.169 *		

* *p* < 0.05; ** *p* < 0.01; *** *p* < 0.001.

**Table 5:** Summary of linear regression analysis for politics and attitudinal factors predicting COVID-19 vaccination rate (N = 74).

	Model 1			Model 2		
Variable	B	SE B	β	B	SE B	β
Government effectiveness	8.107	3.426	0.303 **	6.615	3.816	0.248
Trust science	0.533	0.187	0.290 **	0.384	0.178	0.209 **
Fiscal decentralization index	30.237	13.398	0.264 **	13.839	12.968	0.121
Parliamentary voter turnout 2016–2020 (%VAP)				0.515	0.135	0.321 ***
Corruption perception index				−0.073	0.168	−0.055
Freedom score				0.114	0.082	0.127
R^2^	0.541			0.633		
F for change in R^2^	27.504 ***			5.597 **		

Dependent variable: persons fully vaccinated per 100. ** *p* < 0.01; *** *p* < 0.001.

## Data Availability

Links or references to publicly archived datasets are detailed in the text; nonpublic data used may be requested from the authors.

## Supplementary Materials

The following supporting information can be downloaded at: https://www.mdpi.com/article/10.3390/vaccines10081343/s1, Table S1: Countries Included for the Analysis of the Resources and Political and Attitudinal Models.
