# Supplementary material for: Community Health Resources, Globalization, Trust in Science, and Voting as Predictors of COVID-19 Vaccination Rates: A Global Study with Implications for Vaccine Adherence
Source: Vaccines (Basel). 2022 Aug 18;10(8):1343. doi: 10.3390/vaccines10081343 (PMC9416245; doi:10.3390/vaccines10081343)
Supplement: Supplementary file 1 [file vaccines-10-01343-s001.zip › vaccines-1866471-supplementary.pdf]

**Table S1:** Countries Included for the Analysis of the Resources and Political and Attitudinal Models

| *Country* | *Continent* | *Population 2021* | *Number of fully vaccinated persons per 100 of each country* |
|---|---|---|---|
| *Afghanistan (R)* | Asia | 39835.428 | 11.999 |
| *Algeria (R+P&A)* | Africa | 44616.624 | 14.78 |
| *Angola (R)* | Africa | 33933.61 | 19.253 |
| *Argentina (R+P&A)* | South America | 45605.826 | 82.358002 |
| *Australia (R+P&A)* | Australia | 25788.215 | 84.427002 |
| *Barbados (R)* | Caribbean | 287.711 | 52.863998 |
| *Bangladesh (R+P&A)* | Asia | 166303.498 | 70.694 |
| *Bolivia (R+P&A)* | South America | 11832.94 | 50.695 |
| *Brazil (R+P&A)* | South America | 213993.437 | 74.742996 |
| *Bulgaria (R+P&A)* | Europe | 6896.663 | 29.584 |
| *Burkina Faso (R+P&A)* | Africa | 21497.096 | 7.3649998 |
| *Burma/ Myanmar (R+P&A)* | Asia | 54806.012 | 43.146999 |
| *Cambodia (R+P&A)* | Asia | 16946.438 | 84.981003 |
| *Cameroon (R+P&A)* | Africa | 27224.265 | 4.5549998 |
| *Canada (R+P&A)* | North America | 38067.903 | 82.518997 |
| *Chad (R)* | Africa | 16914.985 | 12.709 |
| *Chile (R+P&A)* | South America | 19212.361 | 91.314003 |
| *China (R)* | Asia | 1444216.107 | 85.446999 |
| *Colombia (R+P&A)* | South America | 51265.844 | 69.589996 |
| *Costa Rica (R+P&A)* | Central America | 5139.052 | 80.178001 |
| *Cuba (R+P&A)* | Caribbean | 11317.505 | 87.805 |
| *Czech Republic (R+P&A)* | Europe | 10724.555 | 64.156998 |
| *Denmark (R+P&A)* | Europe | 5813.298 | 82.207001 |
| *Democratic Republic of Congo (R+P&A)* | Africa | 92377.993 | 1.189 |
| *Dominican Republic* | Caribbean | 10953.703 | 54.948002 |
| *Ecuador (R+P&A)* | South America | 17888.475 | 78.762001 |
| *Egypt (R+P&A)* | Africa | 104258.327 | 33.695 |
| *El Salvador (R+P&A)* | Central America | 6518.499 | 66.114998 |
| *Ethiopia (R+P&A)* | Africa | 117876.227 | 18.52 |
| *Finland (R+P&A)* | Europe | 5548.36 | 78.394997 |
| *France (R+P&A)* | Europe | 65426.179 | 80.230003 |

| | | | |
|---|---|---|---|
| *Germany (R+P&A)* | Europe | 83900.473 | 76.311996 |
| *Ghana (R+P&A)* | Africa | 31732.129 | 19.408001 |
| *Greece (R+P&A)* | Europe | 10370.744 | 71.838997 |
| *Guatemala (R)* | Central America | 18249.86 | 34.173 |
| *Guinea (R+P&A)* | Africa | 13497.244 | 19.118 |
| *Haiti (R)* | Caribbean | 11541.685 | 1.104 |
| *Hungary (R+P&A)* | Europe | 9634.164 | 62.805 |
| *India (R+P&A)* | Asia | 1393409.038 | 62.566002 |
| *Indonesia (R+P&A)* | Asia | 276361.783 | 60.48 |
| *Iran (R+P&A)* | Asia | 85028.759 | 68.567001 |
| *Iraq (R+P&A)* | Asia | 41179.35 | 18.407 |
| *Ireland (R+P&A)* | Europe | 4982.907 | 80.316002 |
| *Israel (non-Palestine territories) (R+P&A)* | Asia | 8789.774 | 73.681999 |
| *Italy (R+P&A)* | Europe | 60367.477 | 79.526001 |
| *Jamaica (R+P&A)* | Caribbean | 2973.463 | 23.152 |
| *Japan (R+P&A)* | Asia | 126050.804 | 80.220001 |
| *Kazakhstan (R+P&A)* | Asia | 18994.962 | 53.776001 |
| *Kenya (R+P&A)* | Africa | 54985.698 | 15.465 |
| *Lebanon (R+P&A)* | Asia | 6769.146 | 34.449001 |
| *Madagascar (R+P&A)* | Africa | 28427.328 | 4.178 |
| *Malawi (R)* | Africa | 19647.684 | 4.75 |
| *Malaysia (R)* | Asia | 32776.194 | 82.472 |
| *Mali (R+P&A)* | Africa | 20855.735 | 5.309 |
| *Mexico (R+P&A)* | North America | 130262.216 | 62.007 |
| *Morocco (R+P&A)* | Africa | 37344.795 | 63.360001 |
| *Mozambique (R+P&A)* | Africa | 32163.047 | 38.243999 |
| *Nepal (R+P&A)* | Asia | 29674.92 | 67.255997 |
| *Netherlands (R+P&A)* | Europe | 17173.099 | 71.258003 |
| *New Zealand (R+P&A)* | The Pacific Ocean | 4860.643 | 84.446999 |
| *Nicaragua (R+P&A)* | Central America | 6702.385 | 70.509003 |
| *Niger (R+P&A)* | Africa | 25130.817 | 6.4000001 |
| *Nigeria (R+P&A)* | Africa | 211400.708 | 7.7280002 |
| *Norway (R+P&A)* | Europe | 5465.63 | 75.210999 |
| *Pakistan (R+P&A)* | Asia | 225199.937 | 54.462002 |
| *Panama (R)* | Central America | 4381.579 | 71.250999 |

| | | | |
|---|---|---|---|
| *Paraguay (R)* | South America | 7219.638 | 47.284 |
| *Peru (R+P&A)* | South America | 33359.418 | 81.447998 |
| *Philippines (R+P&A)* | Asia | 111046.913 | 62.463001 |
| *Poland (R+P&A)* | Europe | 37797.005 | 59.139 |
| *Portugal (R+P&A)* | Europe | 10167.925 | 85.746002 |
| *Romania (R)* | Europe | 19127.774 | 41.969002 |
| *Russia (R+P&A)* | Europe/Asia | 145912.025 | 50.223 |
| *Rwanda (R+P&A)* | Africa | 13276.513 | 64.894997 |
| *South Africa (R)* | Africa | 60041.994 | 30.164 |
| *Saudi Arabia (R)* | Asia | 35340.683 | 71.239998 |
| *Senegal (R)* | Africa | 17196.301 | 43.629002 |
| *Somalia (R)* | Africa | | 8.7150002 |
| *Spain (R)* | Europe | 46745.216 | 78.759003 |
| *Sri Lanka (R+P&A)* | Asia | 21497.31 | 67.513 |
| *Sudan (R+P&A)* | Africa | 44909.353 | 8.2819996 |
| *Sweden (R)* | Europe | 10160.169 | 71.448997 |
| *Tanzania (R+P&A)* | Africa | 61498.437 | 5.8060002 |
| *Thailand (R+P&A)* | Asia | 69950.85 | 73.386002 |
| *Trinidad and Tobago (R+P&A)* | Caribbean | 1403.375 | 50.816002 |
| *Tunisia (R)* | Africa | 11935.766 | 53.875999 |
| *Turkey (R+P&A)* | Europe/Asia | 85042.738 | 63.766998 |
| *Uganda (R+P&A)* | Africa | 47123.531 | 23.105 |
| *UK (R+P&A)* | Europe | 68207.116 | 73.017998 |
| *Ukraine (R)* | Europe | 43466.819 | 34.650002 |
| *USA (R+P&A)* | North America | 332915.073 | 65.556 |
| *Uzbekistan (R+P&A)* | Asia | 33935.763 | 43.478001 |
| *Venezuela (R+P&A)* | South America | 28704.954 | 50.243999 |
| *Zambia (R)* | Africa | 18920.651 | 12.693 |
| *Zimbabwe (R+P&A)* | Africa | 15092.171 | 25.409 |

R = Resource models; P&A = Political & Attitudinal models
